# Genome-Wide Characterization of WRKY Transcription Factors Revealed Gene Duplication and Diversification in Populations of Wild to Domesticated Barley

**DOI:** 10.3390/ijms22105354

**Published:** 2021-05-19

**Authors:** Jinhong Kan, Guangqi Gao, Qiang He, Qian Gao, Congcong Jiang, Sunny Ahmar, Jun Liu, Jing Zhang, Ping Yang

**Affiliations:** Institute of Crop Sciences, Chinese Academy of Agricultural Sciences (CAAS), Beijing 100081, China; kanjinhong@caas.cn (J.K.); gaoguangqi95@163.com (G.G.); qiangh@163.com (Q.H.); dmb_gaoqian@163.com (Q.G.); jiangcongcong@caas.cn (C.J.); sunnyahmar13@gmail.com (S.A.); liujun@caas.cn (J.L.); zhangjing03@caas.cn (J.Z.)

**Keywords:** barley, WRKY transcription factors, diversity, domestication, adaptation

## Abstract

The WRKY transcription factors (WRKYs) are known for their crucial roles in biotic and abiotic stress responses, and developmental and physiological processes. In barley, early studies revealed their importance, whereas their diversity at the population scale remains hardly estimated. In this study, 98 *HsWRKYs* and 103 *HvWRKYs* have been identified from the reference genome of wild and cultivated barley, respectively. The tandem duplication and segmental duplication events from the cultivated barley were observed. By taking advantage of early released exome-captured sequencing datasets in 90 wild barley accessions and 137 landraces, the diversity analysis uncovered synonymous and non-synonymous variants instead of loss-of-function mutations that had occurred at all *WRKYs*. For majority of *WRKYs*, the haplotype and nucleotide diversity both decreased in cultivated barley relative to the wild population. Five *WRKYs* were detected to have undergone selection, among which haplotypes of *WRKY9* were enriched, correlating with the geographic collection sites. Collectively, profiting from the state-of-the-art barley genomic resources, this work represented the characterization and diversity of barley WRKY transcription factors, shedding light on future deciphering of their roles in barley domestication and adaptation.

## 1. Introduction

The cultivated barley (*Hordeum vulgare* subsp. *vulgare*, *Hv*) is the fourth important cereal crop on yield production and cultivation area globally (FAO dataset, 2019), which was domesticated approximately 10,000 years ago from its wild progenitor *Hordeum vulgare* subsp. *sponteneum* (*Hs*) [1,2]. Both wild and cultivated barley belong to the same species without crossing a barrier. Nowadays, two-thirds of its grain production is used for animal feed and the remaining portion for malting and brewing industries, as well as the staple food in the Himalayas area and some African countries [3]. In cultivated barley, the six-rowed spike with non-brittle rachis and hulless caryopses resulted from the simultaneous selection of non-functional alleles at the three loci, namely *Vrs1* [4], *Btr1* (or *Btr2*) [5] and *Nud* [6]. *Vrs1* and *Nud* encode transcription factor of homeodomain-leucine zipper (HD-ZIP) and ethylene responsive factor (ERF), respectively. The locus *Vrn1*, which controls the vernalization process and promotes the flowering in autumn-sowing barley [7], was identified to encode a MADS box transcription factor [8]. The transcription factors played important roles in shaping of the population diversity and extending of the adaptation of cultivated barley, as were the cases that have been demonstrated in other species [9,10].

The WRKY transcription factors represent a large family of transcriptional regulators in higher plants. The family members harbor the WRKY domain(s) that specifically binds to the *cis*-acting motif W-box (C/TTGACT/C) in the promoter regions, to initiate either activation or repression of their target genes’ expression [11,12]. The WRKY domain consists of approximately 60 amino acid residues, including a conserved WRKYGQK motif at the N’-terminal and a C_2_H_2_- or C_2_HC-type zinc finger motif at the C’-terminal [13,14]. The members of the WRKY family were categorized into three groups based on the number of WRKY domains and the type of zinc fingers. The proteins within Group I contain two WRKY domains, whereas Group II and III have a single WRKY domain harboring a C_2_H_2_ and C_2_HC zinc finger motif, respectively [13]. WRKYs within Group II were further assigned into five sub-groups (namely IIa, IIb, IIc, IId and IIe) according to constitutes of the conserved amino acids [13]. As a large family of genes, 74 WRKY members in *Arabidopsis* [15,16], 125 in rice (*Oryza sativa*) [17], 171 in common wheat (*Triticum aestivum* L.) [18], 133 in soybean (*Glycine max*) [19] and 136 in maize [20] have been identified.

WRKYs have been unraveled, involved in a broad range of biological processes, such as embryogenesis [21], seed germination [22], lateral root formation and growth [23,24], stem elongation [25,26], trichome development [27], flowering [28], starch synthesis [29], seed development [30], fruit ripening [31], leaf senescence [32,33] and secondary metabolic processes [34,35]. In addition, WRKYs play crucial roles in biotic/abiotic responses [14,36,37,38,39]. For example, three WRKYs (*AtWRKY18*, *40*, *60*) negatively modulated the basal defense against the bacterial pathogen *Pseudomonas syringae* and the fungus *Golovinomyces orontii* [40,41,42,43], while *AtWRKY33* and *AtWRKY57* played contrary roles in regulating abscisic acid (ABA) or jasmonic acid (JA)-mediated signaling pathways under necrotrophic fungus *Botrytis cinerea* infection [44,45,46]. Besides, WRKYs have also been reported to be extensively responsive to abiotic stresses such as nutrient deficiency, drought, waterlogging, salinity, temperature stress and oxidative stress [38].

In cultivated barley, 94 *WRKY* genes have been reported based on the transcripts database or the physical map [47,48,49]. Among these genes, *WRKY1*, *WRKY2* and *WRKY3* could negatively regulate the innate immunity against the powdery mildew fungus *Blumeria gramminis* f. sp. *Hordei* (*Bgh*), by attenuating MLA (Mildew A)-dependent effector-triggered immunity [50,51]. The *WRKY1* and *WRKY2* could also repress the activity of a germin-like defense-related protein HvGER4c, which was induced by *Bgh* [49]. *WRKY10*, *19* and *28* played positive roles in basal resistance and MLA-mediated immunity [48,50,52], and *WRKY6, 40* and *70* were involved in *NPR1* (*Non-expressor of Pathogenesis-Related genes 1*)-mediated acquired resistance [53]. In addition, *WRKY23* improved the resistance to *Fusarium graminearum* through regulating the production of secondary metabolites [54]. Moreover, several *WRKYs* were inducible upon abiotic stresses [55,56,57], amongst which the constitutive expression of *HvWRKY1* significantly improved the viability and drought tolerance [58].

Barley is a diploid inbreeding species with a relatively simple genome, which makes it a model for the genetics and genomics studies in *Triticeae* [59]. This study carried out a genome-wide characterization of *WRKY* genes by using the latest reference genome of the cultivated barley ‘Morex’ [60] and the fragmented draft genome of the wild barley ‘AWCS276’ [61], followed by the sequence diversity analysis among 90 geographically referenced wild barley accessions and 137 landraces [62]. The subcellular localization and transcriptional activation assay were conducted to identify the possible diversification among different haplotypes of *HvWRKY9*. This work provided insights for future characterization of WRKY family members, and implied that the *WRKYs* might be a target for barley improvement.

## 2. Results

### 2.1. Genome-Wide Identification of WRKYs in Wild and Cultivated Barley

The barley WRKY family genes have been characterized by the use of transcribed sequences or the physical map [47,48,49]. However, a reference genome-based characterization of *WRKY* genes remained to be conducted. Here, by taking advantage of the barley reference genomes [60,61], an in silico prediction using PlantTFDB revealed 98 and 101 *WRKY*-like sequences in wild and cultivated barley, respectively (Figure S1). Moreover, searching for homolog sequences of the identified *WRKYs* in *Arabidopsis thaliana* and *Oryza sativa* [15,16,17,63] revealed 73 and 89 WRKY candidates from wild and cultivated barley, respectively (Supplementary Figure S1); however, they were all represented in the *WRKY*-like sequences revealed by PlantTFDB. Following the confirmation of the WRKY domain with HMM and CDD tools, 98 and 101 WRKY domain-containing genes were identified in wild and cultivated barley, respectively.

We further checked redundancy of the 101 WRKY domain-containing genes in cultivated barley, against the 94 *WRKYs* that were reported previously [49]. There were 87 *WRKYs* shared, whereas 14 new *HvWRKY*-like members have been identified in this study (Figure S1). Notably, we identified three replicates (*HvWRKY35* vs. *41*, *49* vs. *72*, *55* vs. *87*), which were reported in the previous study [49], that were shown to be only three genes involved in alternative splicing. The formerly identified genes *HvWRKY16* and *HvWRKY25* were discarded because of deficiency in complete coding frame. *HvWRKY55*/*87* and *HvWRKY77* were not found in the annotated gene list [60], however both were qualified considering an intact coding frame with a WRKY domain and a homologous sequence found from ‘Morex_v2’. Therefore, 103 *WRKYs* from cultivated barley (named *HvWRKYs*) were ultimately identified based on the latest reference genome (Table S1). The additional *WRKYs* newly identified in this study were designated *HvWRKY96* to *HvWRKY109*.

Furthermore, 98 *WRKY*-like sequences in wild barley were subjected to BlastP against the 103 *HvWRKYs*. Ninety-one were found with orthologous genes in cultivated barley (*HsWRKY1* to *HsWRKY109*) (Table S2), while seven without orthologous were named *HsWRKY110* to *HsWRKY116*. Collectively, 98 *HsWRKYs* from wild barley were finally identified.

### 2.2. Classification and Domain Composition of WRKY Proteins

**Figure 1:**
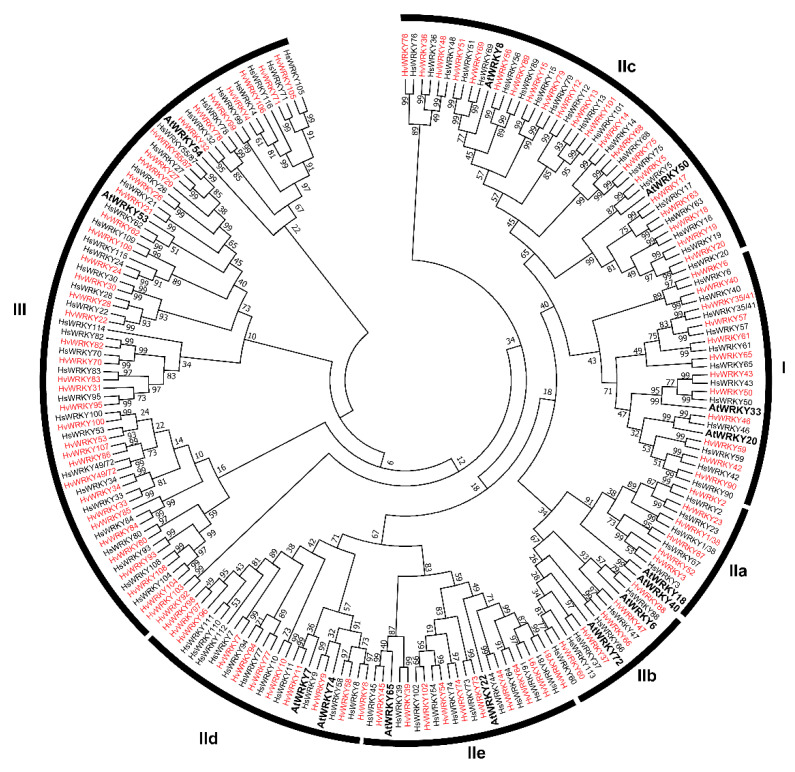
The phylogenetic tree of barley *WRKYs*. The *WRKYs* derived from wild and cultivated barley are highlighted with black and red font respectively, while *A. thaliana WRKYs* is represented with bold black. The values of the nodes are the bootstraps. The groups and sub-groups are indicated (I, II a–e and III).

Making use of 201 WRKYs from barley together with 14 WRKYs of *A. thaliana* from phylogenetic groups/sub-groups [15,16], an unrooted maximum-likelihood phylogenetic tree was generated (Figure 1). Three major groups (I, II and III) have been classified according to the diversification on the number of WRKY domain and the types of the zinc fingers. In Group I, which contained two C_2_H_2_-type WRKY domains, 14 and 13 WRKYs from wild and cultivated barley were assigned, respectively. Fifty-three HsWRKYs as well as HvWRKYs with a single C_2_H_2_-type WRKY domain were assigned to Group II. The remaining 31 HsWRKYs and 37 HvWRKYs which carry the C_2_HC-type WRKY domain were assigned to Group III. HsWRKY24, HsWRKY116 and HvWRKY24 were still classified into Group I, owing to two WRKY domains present, although their C-terminal domain belongs to the C_2_HC-type WRKY domain and is clustered in Group III in the phylogenetic tree. The WRKYs within Group II were further clarified into five subgroups (IIa, IIb, IIc, IId and IIe) based on the feature of conserved amino acid sequences other than in WRKY domains. Besides the highly conserved WRKYGQK motif in the WRKY domain, seven variants were found (Tables S1, S2). In addition to WRKY domains, several other domains were detected (Table S3). For example, both NB-ARC (pfam00931) and Rx_N (pfam18052) domains in HsWRKY24 and HvWRKY24 were known with the feature of disease resistance protein, as is the case of *Arabidopsis* WRKY52/RRS1 which was able to recognize the pathogenic effectors [14,64]. Moreover, the VQ (pfam05678) domain, which is present in VQ proteins and interacts with WRKY proteins to possibly offer plant immunity [65], was found in HsWRKY115 of wild barley.

**Figure 2:**
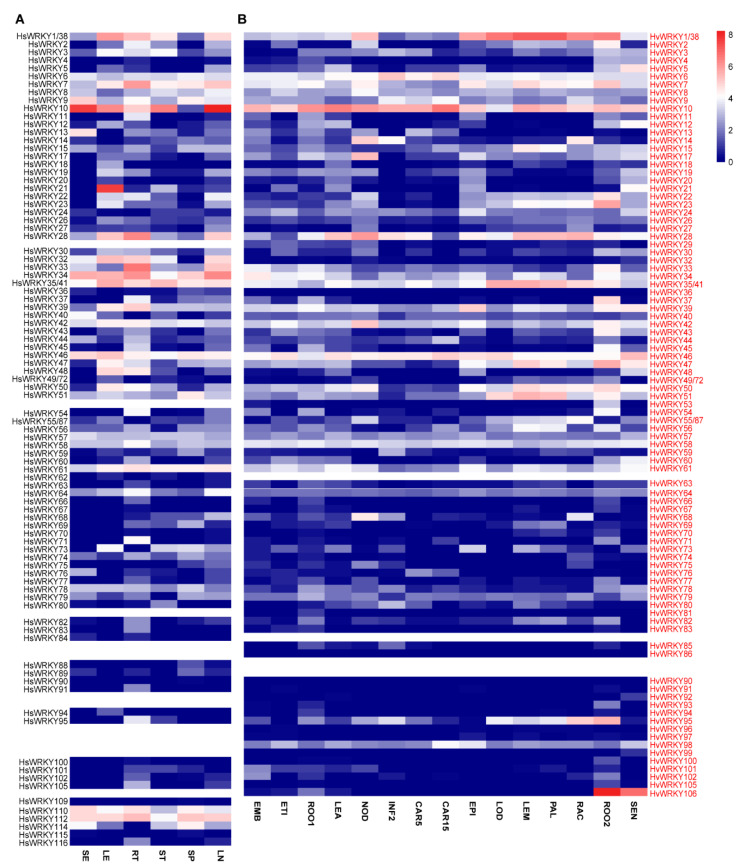
Transcriptional profiles of *HsWRKY* and *HvWRKY* genes. The expressional profile of *WRKYs* in different tissues from the wild (**A**) and cultivated (**B**) barley. SE, whole seedlings of 15 days after planting (dap); LE, leaves from seedlings at 25 dap; RT, roots collected at 30 dap; ST, first stems dissected at 42 dap; SP, spikelets obtained at anthesis; LN, developing kernels collected at 15 days post-anthesis. EMB, 4-day embryos; ETI, etiolated seedling at 10 dap, dark condition; ROO1, roots from seedlings (10 cm shoot stage); LEA, shoots from seedlings (10 cm shoot stage); NOD, developing tillers, 3rd internode at 42 dap; INF2, developing inflorescences (1–1.5 cm); CAR5, developing grain at 5 days post-pollination (dap); CAR15, developing grain (15 dap); EPI, epidermal strips (28 dap); LOD, inflorescences, lodicule (42 dap); LEM, inflorescences, lemma (42 dap); PAL, dissected inflorescences, palea (42 dap); RAC, inflorescences, rachis (35 dap); ROO2, roots (28 dap); SEN, senescing leaves (56 dap). Heatmap was generated based on logarithm values of FPKM and the colored scale for the expression levels is shown.

To dissect the transcriptional pattern of individual *WRKY* genes in wild and domesticated barley, we further analyzed the transcriptional profiles using the released RNA-seq datasets [61,66]. Out of those *WRKY* genes, 89 *HsWRKYs* (Figure 2A) and 91 *HvWRKYs* (Figure 2B) were detected with transcripts in wild and cultivated barley, respectively. Similarity on the transcriptional pattern in each of the orthologous genes between wild and cultivated barley was detected. For example, *HsWRKY10* and *HvWRKY10* were found to be highly expressed in all tissues, in contrast with the *HsWRKY90* and *HvWRKY90* that exhibited very low abundance. There are some *WRKYs* that were highly expressed in particular tissues, such as *HsWRKY21* with a higher expression was detected in leaves of wild barley. Moreover, *HvWRKY4*, *HvWRKY105* and *HvWRKY106* were segmentally duplicated genes, whereas *HvWRKY106* was detected with a much higher transcriptional level than that of *HvWRKY4* and *HvWRKY105* in roots (ROO2) and senescing leaves (SEN). Whether the gene duplication was relevant with re-programmed transcription or specified function in particular tissues is worth further investigation.

### 2.3. The Duplication Events in Barley HvWRKYs

Gene duplication is a main driving force along evolution, which creates the raw genetic materials for natural selection [67], and also results in the expansion of gene families [68]. The duplication analysis was feasible with 103 *HvWRKY*s, whereas infeasible with *HsWRKYs* which lack the linearized chromosome (Table S2). We identified seven segmental duplication events that corresponded to 17 *HvWRKYs* (Figure S2, Table S4). Besides, six tandem duplication clusters consisting of 14 *HvWRKYs* were detected on five chromosomes of the ‘Morex’ genome (Figure S2). On the long arm of chromosome 1H, there was an enrichment of *HvWRKY*s in two tandem duplication clusters, including six *HvWRKYs* which were located. Interestingly, eight out of twelve genes that are absent in wild barley but present in cultivated barley were duplicated genes. Several *HvWRKYs* (i.e., *HvWRKY4*, *71*, *105*, *106*) were found having multiple duplicates. Collectively, the duplication of *WRKY* genes has possibly been involved in the differentiation of wild and cultivated barley.

### 2.4. Sequence Diversity of WRKYs in Wild and Cultivated Barley

We further analyzed the genetic diversity of 103 *WRKY* genes at the population scale to identify whether *WRKYs* are involved in barley domestication or local adaptation to adverse conditions. This collection includes 90 wild barley accessions mainly from the Fertile Crescent and Central Asia, and 137 landraces from Europe, Asia and Africa (Table S5). Out of 103 *WRKYs*, 37 genes were excluded due to high missing ratio in the population, while 66 were qualified for the following analysis. Remarkably, no variants encoding a loss-of-function (*LoF*) protein were detected at any loci (Table S6). It suggested that *WRKYs* were essential in both wild and cultivated barley. All *WRKYs*, except *WRKY4* and *WRKY64*, were found with the same or a decrease in the number of haplotypes in landraces vs. wild barley population. Meanwhile, both haplotype diversity (*H*) and nucleotide diversity (*π*) in the majority of the 66 *WRKYs* counterparts decreased in landraces relative to the wild barley population (Table S6).

**Figure 3:**
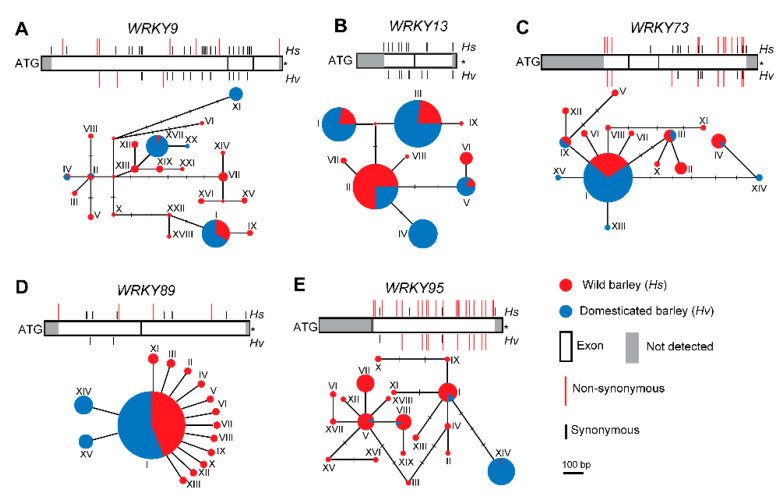
Haplotype analysis of nine *WRKYs* in wild and cultivated barley population. The nucleotide substitutions in the coding region and the haplotype network of *WRKY9* (**A**), *WRKY13* (**B**), *WRKY73* (**C**)*, WRKY89* (**D**) and *WRKY95* (**E**). The rectangles represent the coding region of each gene, while the grey box indicates the part without qualified sequences. The size of each circle briefly refers to the number of the accessions containing a particular haplotype. Each short solid line represents a single mutation among haplotypes. The red and black lines indicate the positions of non-synonymous and synonymous mutations in the coding sequence, respectively. ^*^ Stop codon removed, *Hs*, *Hordeum vulgare ssp. spontaneum*; *Hv*, *Hordeum vulgare ssp. vulgare*.

In order to analyze *WRKY* genes under selection, we calculated genome-wide Tajima’s *D* and found the empirical distribution of Tajima’s *D* in either wild (range: –2.52 to −5.03) or cultivated barley population (range: −2.68 to 5.53) (Figure S3). We chose 2.5% and 97.5% quantiles of Tajima’s *D* distribution of each barley compartment as thresholds for detecting *WRKY* genes with selection signature. Eleven *WRKYs* with the significant selection signal were identified, whereas only five *WRKYs* (*WRKY9*, *WRKY13*, *WRKY73*, *WRKY89* and *WRKY95*) qualified (Table S6). *WRKY89* in wild barley population was detected with the selection signal, while the remaining four *WRKYs* were found in cultivated barley population (Figure S3). We further built up the polymorphism and the haplotype networks at these five *WRKYs* (Figure 3A–E). For example, 15 haplotypes of *WKRY89* were detected in wild and cultivated barley in all, amongst which hap-I was found with an enrichment in wild barley population (78.9%, 71 of 90) (Figure 3D, Table S5), and was maintained with a considerable proportion in landraces (65.7%, 90/137) (Figure 3D, Table S5). The landraces carrying this haplotype had a wide range of the geographical distribution (5.63N to 61.78N; 17.81W to 142.64E) (Table S5).

### 2.5. Selection of WRKY9 in Wild and Cultivated Barley

**Figure 4:**
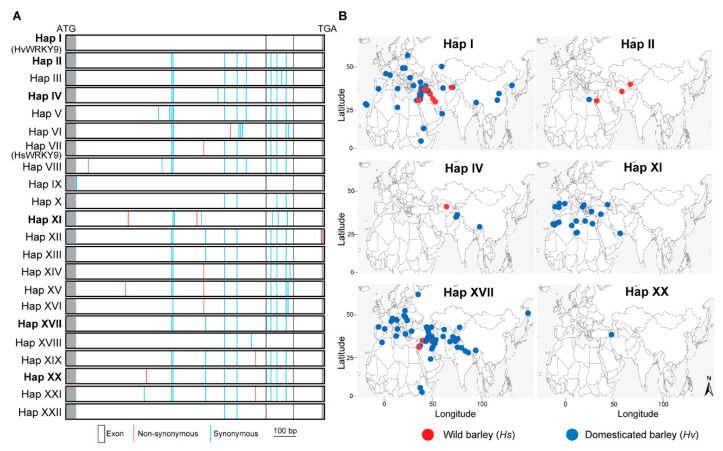
Haplotypes of *WRKY9* and their geographical distribution. (**A**) Graphical representation of full-length coding region in each haplotype. The non-synonymous and synonymous substitutions if compared to Hap I (*HvWRKY9*) were indicated using red and blue lines. Hap VII showed a sequence identical to *HsWRKY9* identified from the reference assembly of wild barley ‘AWCS276’. The bold-highlighted haplotypes were detected in cultivated barley. The red and blue lines indicated non-synonymous and synonymous, respectively. The rectangles represent the coding region of each haplotype, while the grey box indicates the part without qualified sequences. (**B**) Geographical distribution of barley accessions harboring respective haplotypes. Each accession is allocated according to latitude and longitude coordinates of its collection site. The red and blue circles indicate the wild and cultivated barley accessions, respectively.

From the diversity analysis as described above, *HvWRKY9* was the one that showed the highest positive value of Tajima’s *D* in landraces, fitting a model of post-domestication selection. The 1130 bp valid sequences from 71 wild barley accessions and 119 landraces were analyzed, therefore 20 and 6 haplotypes were identified within wild and cultivated barley, respectively (Figure 4A, Table S5). The four haplotypes (hap-I, II, IV, XVII) were shared between wild and cultivated barley (Figure 3A), while two (hap-XI and hap-XX) were only present in landraces and nineteen were exclusively found in wild barley. Haplotypes I and XVII were predominant, accounting for 31.6% (60/190) and 28.9% (55/190) of the collection, respectively (Table S7). Both haplotypes originated from wild barley and were inherited in landrace populations with a range of geographical regions (5.63N to 61.78N; 17.81W to 61.78E), including the barley diversity centers Near East/Europe, East Asia and Ethiopia [69] (Figure 4B). Haplotype XI presenting in 11.6% of the collection was extensively found from the landraces surrounding the Mediterranean Sea (Figure 4B), where the climate was mild and rainy in winter.

**Figure 5:**
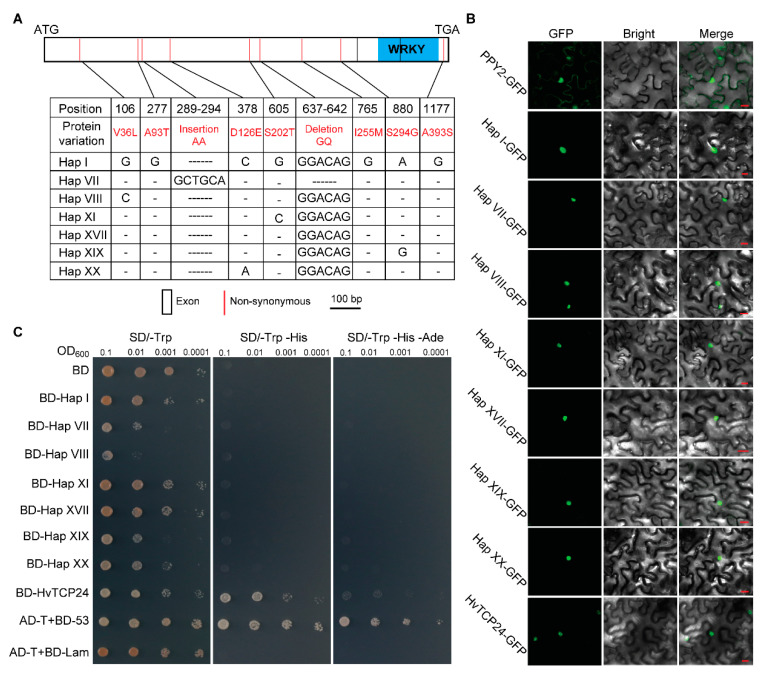
The subcellular localization and transactivation activity of *WRKY9* haplotypes. (**A**) Graphical representation of the full-length coding sequence of selected haplotypes. Only non-synonymous substitutions are indicated. The blue rectangle represents the WRKY domain. (**B**) Subcellular localization of *WRKY9* haplotypes in *N. benthamiana*. Scale bar = 20 μm. (**C**) Transactivation activity of *WRKY9* haplotypes in yeast. BD-HvTCP24, which contains a transactivation activity in yeast, serves as a positive control. SD, synthetic dextrose medium; Trp, tryptophan; His, histidine; Ade, Adenine. 0.1, 0.01, 0.001 and 0.0001 denoted the different dilution series.

To investigate whether the haplotype diversification was associated with a biological relevance (e.g., the binding activity), seven *HvWRKY9* haplotypes were examined for their subcellular localization in *Nicotiana benthamiana* and the transactivation activities in yeast. These haplotypes, including the major haplotypes I and XVII (both encoding an identical amino acid sequence) and five minor haplotypes (VII, VIII, XI, XIX, XX), represented different constitutes on the amino acid sequence (Figure 5A). To examine if *WRKY9* haplotypes were speculative with shift on subcellular localization, the full-length coding sequence of each haplotype was fused with a C’-terminal GFP sequence and was subjected to the subcellular localization assay. The fusion protein was found in the nucleus for each of the haplotypes (Figure 5B). Meanwhile, each of the respective *HvWRKY* haplotypes was transformed into yeast strain Y2HGold, followed by a selection on the amino acid-deficient media. However, there was no transactivation activity observed for any of the *HvWRKY9* haplotypes (Figure 5C).

## 3. Discussion

In this study, we performed the genome-wide identification and diversity analysis of *WRKYs* within wild and cultivated barley using the latest reference genomes [60,61]. In comparison with the previous studies based on data resources from the transcriptome or the physical map [47,48,49], the high-quality reference genome from the cultivated barley (Morex_v2) enabled the identification of 103 high-confidence *WRKYs*, notably including 14 members that had not been reported (Table S1). For example, three pairs of putative genes detected from the transcriptome were shown to be only three genes involved in alternative splicing, and two pseudogenes with a pre-stop codon were identified. Moreover, 98 *WRKYs* from the wild barley have been identified for the first time (Figure 1). Ninety-one *WRKYs* were conserved on sequence identity between wild and cultivated barley (Figure 1). Notably, there were seven and twelve *WRKYs* exclusively found in wild and cultivated barley, respectively (Figure 1). The divergence might be due to: (1) the imperfect drafted wild barley genome [61], (2) incorrect gene annotation (i.e., two genes, *HvWRKY55*/*87* and *HvWRKY77,* were absent in the annotated gene list), (3) varied gene constitution between particular genotypes of either wild or cultivated barley and (4) the gene duplication during barley domestication. For the future perspective, the availability of a barley pan-genome would help to better decipher the diversification of barley *WRKYs* [70,71]. Moreover, this study identified a series of *HvWRKY*s in tandem or segmental duplications (Figure S2), like the cases characterized in other species. The tandem and segmental duplications involving 5 and 80 *WRKY* genes respectively, have been reported in wheat [18], and in white pear, 33 and 57 *PbWRKYs* were tandemly or segmentally duplicated [72]. The duplication is therefore one of the driving forces that contributes to the expansion of the *WRKY* gene family. We found it was interesting that HsWRKY24, HsWRKY116 and HvWRKY24 harbor two WRKY domains, however these three proteins have been clustered with WRKYs from group III based on the C-terminal C_2_HC-type WRKY domain, which have been reported in *Saccharum spontaneum* [73]. It supports the speculation that some WRKY I proteins evolved from the duplication of individual domains on the group III WRKYs in Gramineae [74].

In addition to the WRKY domain, the identified WRKY proteins represented several domains, such as NBS-LRR or VQ (Table S3). Three *WRKYs* encoded for chimeric proteins assembled by an NBS-LRR domain and the WRKY domain(s). In *A. thaliana*, the AtWRKY52/RRS1, a chimeric protein harboring an NBS-LRR domain, is a receptor that recognizes pathogenic effectors [75] and activates immune responses [76]. The *HsWRKY115* included a VQ motif, which was specifically identified in plants and may interact with other WRKY transcription factors and/or MAPKs to regulate plant defense, growth and development processes [77,78,79,80]. WRKY TFs have played important roles in responding to abiotic and biotic stresses [14,36,37,38,39], whereas the abundant genetic diversity has benefited adaptations to the adverse environments [81]. This study evaluated the diversity of *WRKY* genes in wild and cultivated barley populations by taking advantage of exome-captured sequencing data source [62]. The haplotype diversity and the nucleotide diversity at the majority of the *WRKYs* reduced from wild to cultivated barley population (Table S6). There might be few haplotypes (i.e., hap-I of *WRKY89*, which presented in 90 out of 137 barley landraces) that are adaptable under various environmental conditions and that have been multiplied from the wild progenitors, therefore becoming predominant in the cultivated barley population (Figure 3D). For *HvWRKY1* (referring to *HvWRKY1/38* from this study) and *HvWRKY2*, which were identified by screening of the cDNA library with MLA receptor as bait and acted as repressors in fungus infection [49,51] as well as played positive roles in response to abiotic stresses [55,56,58], there was no significant selection detected within wild or cultivated barley populations. Collectively, this result provided insights on the diversification of barley *WRKY* family members and would support the future deciphering of *WRKYs’* function in biotic and abiotic stresses responses.

*HvWRKY9* had the highest positive Tajima’s *D* value in barley landrace population. The haplotypes XI and XX were exclusively found in cultivated barley (22 and 1 out of 137, respectively) (Figure 3A), while hap-XI was enriched surrounding the Mediterranean Sea (Figure 4A, B). This region at the lower latitude generally represents a mild and rainy climate over winter. In *Arabidopsis*, *AtWRKY39*, the ortholog of *HvWRKY9*, was inducible under heat stress [82]. Accordingly, *HvWRKY9*, especially the haplotype XI, might also play roles in adaptation upon the climatic changes. However, we did not detect any difference among *HvWRKY9* haplotypes on their subcellular localization in *N. benthamiana* (Figure 5B) or transactivation activity in yeast (Figure 5C), which indicated that the diversification among *HvWRKY9* haplotypes was rather the modification on protein subcellular retention or the transactivation activity. The sharp decline of the nucleotide diversity and haplotype diversity of *WRKY9* and other *WRKYs* was identified in barley landrace to wild barley, which was consistent with the theory of a domestication bottleneck [83]. However, in recent research, which showed little change in heterozygosity between archaeological barley and the wild progenitor, a contradiction to the domestication bottleneck was suggested as the cases reported from maize and Sorghum [84,85]. The domestication bottleneck remains evaluated relying on the genomic diversity of barley from different history terms. The secondary substructure (if genetically defined) within wild or domesticated barley is a factor relevant to the detection of selection signatures (e.g., the cases revealed in [86]), and we believe that some signatures of selection are detectable only among geographical genetic groups. However, the crop underwent regionally specific episodes of gene flow, and selection has previously been interpreted as evidence of multiple domestications [87,88]. We attempted to decipher the *WRKY* involved in selection from two main structures (wild barley vs. cultivated barley) [62]. There might be a limit on detection of selection and especially local adaptation (e.g., some *WRKYs* with selection signatures are only detected at genetically defined groups).

## 4. Materials and Methods

### 4.1. Data Resources

The genomic sequences of wild barley accession ‘AWCS276’ (Hs, WB_v0.5) [61] and cultivated barley variety ‘Morex’ (Hv, Morex_v2, 18 November 2019) [60] were obtained from the public database. The protein sequences of WRKYs in *A. thaliana* and *O. sativa* were retrieved from TAIR and the Rice Genome Annotation Project Database based on accession IDs obtained from previous publications, respectively [15,16,17,63]. The exome-captured sequencing datasets of 90 wild barley accessions and 137 landraces which had geographically referenced passport information [62] were downloaded from the NCBI SRA database (project: PRJEB8044/ERP009079).

### 4.2. Identification of WRKYs in Wild and Cultivated Barley

The identification of *WRKY* genes was conducted with the following pipeline. First, the coding sequences of wild and cultivated barley were detected using the Plant Transcription Factors Database v4.0 (PlantTFDB, 24 October 2016) [89], to predict *WRKY*-like sequences. Second, using already published WRKY protein sequences of *A. thaliana* and *O. sativa* as queries, BlastP against the coding sequences of barley genomes was carried out in order to identify *WRKY*-like sequences. Finally, all the predicted sequences were checked for the presence of a functional WRKY domain with HMM Hidden Markov models (HMMs, E-value ≤ 1 × 10^−5^) and the Conserved Domains (CDD) tool [90]. The non-redundant sequence encoding for an intact protein with complete WRKY domain(s) was accepted as WRKYs and designated following the nomenclature pipeline proposed by Liu [49]. The *HvWRKYs*, which were identified from Morex_v2 in this study, were subjected to BlastP against the published barley WRKY protein sequences [49] in order to identify their correspondence with those designated genes. The *HvWRKYs* without a counterpart in the previously identified ones were sequentially designated as *HvWRKY96* to *HvWRKY109*. The *HsWRKYs* from wild barley were designated accordingly based on their sequence homology against the *HvWRKYs* in cultivated barley.

The online software ExPASy [91] was used to analyze the protein properties, such as the length of the protein, molecular weight (MW), theoretical isoelectric point (pI), grand average of hydropathicity (GRAVY), aliphatic index (AI) and instability index (II). The SignalP v5.0 server [92] and TargetP v2.0 server [93] were used to predict the cleavage site of a signal peptide and the subcellular location of the protein, respectively. The chromosomal location of *HvWRKYs* was identified by carrying out BlastN against the Morex_v2 pseudomolecules, followed by a visualization using MapInspect software. A phylogenetic tree was generated by MEGA7 using the maximum likelihood method with the parameters (test of phylogeny, 1000 bootstrap replicates; gaps/missing data treatment, partial deletion; Model/Method, Jones–Taylor–Thornton model; rates and patterns, Gamma distributed with invariant sites). Arabidopsis *WRKYs* from phylogenetic sub-clades [13] were deployed ensuring the reliability of the barley phylogenetic tree.

The criteria used for identifying gene duplication were as follows: (a) the length of aligned region spanned > 75% of the gene sequence, and (b) the aligned region had similarity > 75% between two genes [94]. Two or more adjacent duplicates within 100 kb were considered as tandem duplication [31], while duplicates across different chromosomes or within a distance larger than 100 kb on the same chromosome were defined as segmental duplication [95,96].

### 4.3. Tissue and Temporal Expression

The transcriptional expression of *HsWRKYs* was analyzed in six tissues of ‘AWCS276’ based on the RNA-seq data downloaded from the NCGR database [61]. For the *HvWRKYs* in cultivated barley, their transcriptional profiles in ‘Morex’ were downloaded from BARLEX [66,97]. The heatmap was generated using R language with the pheatmap package, based on means of the logarithm values of fragments per kilobase of transcript per million (FPKM).

### 4.4. Diversity Analysis in Wild and Cultivated Barley Populations

The low-quality reads and adapters from exome-captured sequencing datasets were excluded using *fastp* [98] with the parameters (− *q* = 15; − *u* = 40; − *n* = 5; − *l* = 15). The paired reads were mapped to barley reference genome Morex_v2 [60] using bowtie2 software [99], followed by SNP calling using GATK4 [100] with Russell’s method [62]. For each locus, if the reads number of alternative allele/total depth was below 0.8 or over 0.2, then it was defined as heterozygosity. The haplotype sequences for each sample were generated using Python scripts, by converting SNPs/Indels on the reference sequence at each variation site. The sequence reads were obtained from introns, and those with poor quality or heterozygosity were removed from the following analysis. Sequence assembly and alignments were performed using Sequencher v4.7. When the assembled sequence for a gene locus was less than 100 bp, which is less convincing referring to the gene variation, or the sequences with missing data or heterozygotes, the respective genes were excluded from the analysis. The polymorphic sites that encode for loss-of-function (*LoF*), synonymous (*S*) or non-synonymous (*Ns*) were manually recorded. The haplotypes, the haplotype diversity (*H*) and the nucleotide diversity (*π*) were calculated using DNASP v5.10.01 [101]. The Median-Joining (MJ) network was generated using Network v4.6.1.1, based on the files with polymorphisms exported from DNASP v5.10.01 and DNA alignment v1.3.1.1. The genome-wide Tajima’s *D* in the coding sequences was performed by using VCFtools v0.1.13 [102]. We chose 2.5% and 97.5% quantiles of Tajima’s *D* distribution of each barley compartment as thresholds for detecting *WRKY* genes with significant Tajima’s *D* values (−1.68324 and 2.38961 for cultivars, −1.98658 and 1.82940 for wild barley, respectively). The topographic maps were produced using five packages (maps, sp, map tools, ggplot2 and mapproj) in R language according to geographical information of the barley accessions that carry a particular haplotype.

### 4.5. Subcellular Localization in N. benthamiana

The *HvWRKY9* haplotypes including III, VII, VIII, XI, XVII, XIX and XX were obtained by nucleotide synthesis (Sangon, Shanghai, China). Each haplotype fragment was amplified with gene-specific primers (Table S8), followed by the insertion into the plasmid pDONR207 using the Gateway BP Clonase II Enzyme mix (Thermo Fisher Scientific, Wilmington, DE, USA). The sequence-verified entry vector using Sanger sequencing was recombined with the destination plasmid (pUBC_GFP_DEST) with a C’-terminal green fluorescent protein (GFP) fusion, driven by the Arabidopsis ubiquitin-10 (UBQ10) gene promoter [103]. The plasmid was transformed into *Agrobacterium tumefaciens* strain GV3101 using freeze–thaw methods. The agrobacteria harboring a WRKY-GFP construct were resuspended using infiltration buffer (10 mM 2-(N-morpholino) ethanesulfonic acid (MES), pH 5.7; 10 mM MgCl_2_; 150 μM acetosyringone (AS)), and then infiltrated into four-week-old *N. benthamiana* leaves, followed by imaging with the confocal microscope (Carl Zeiss LSM880, Oberkochen, Germany) on days 2 to 3 post-infiltration.

### 4.6. Transcriptional Activation in Yeast

The transactivation activity assay was conducted in yeast strain Y2HGold. In brief, the pDONR207 entry vector with full-length CDS of five *HvWRKYs* or respective *HvWRKY9* haplotypes was recombined by the LR enzyme into the destination vector GAL4-pGBKT7, which was previously introduced with the Gateway LR recombination sites. The sequence-verified plasmids were transformed into yeast using standard PEG/LiAc transformation methods. The transformants were checked for the plasmid presence on the synthetic dextrose growth medium without amino acid tryptophan (Trp, SD/-Trp), followed by exhibiting the transactivation activity on the synthetic dextrose selection media (SD/-Trp-His, without tryptophan and histidine; SD/-Trp-His-Ade, without tryptophan, histidine and adenine).

## Data Availability

The data presented in this study are available in the manuscript and as supplementary material.

## Supplementary Materials

The following are available online at https://www.mdpi.com/article/10.3390/ijms22105354/s1, Figure S1: The workflow of identification of *WRKYs* in wild and cultivated barley. Figure S2: Chromosomal locations of *HvWRKYs*. Figure S3: Violinplot of the Tajima’s *D* of all genes and *WRKYs* in wild and cultivated barley population. Table S1: The *WRKY* genes identified from cultivated barley, Table S2: The *WRKY* genes identified from wild barley. Table S3: Presence and position of conserved domains identified within barley WRKY proteins using HMMER. Table S4: Tandemly and segmentally duplicated genes identified in *HvWRKYs*. Table S5: Passport information of 227 barley accessions and the haplotypes of five *HvWRKYs*. Table S6: Sequence diversity and selection statistics of *WRKY* genes in wild and cultivated barley populations. Table S7: Information of *HvWRKY9* haplotypes. Table S8: The primers used in this study.
