# Supplementary material for: Genome-Wide Characterization of WRKY Transcription Factors Revealed Gene Duplication and Diversification in Populations of Wild to Domesticated Barley
Source: Int J Mol Sci. 2021 May 19;22(10):5354. doi: 10.3390/ijms22105354 (PMC8160967; doi:10.3390/ijms22105354)
Supplement: Supplementary file 1 [file ijms-22-05354-s001.zip › Table S6-Sequence diversity and selection statistics of WRKY genes in wild and cultivated barley populations.pdf]

Table S6. Sequence diversity and selection statistics of *WRKY* genes in wild and cultivated barley populations

Genes	Gene IDs	Type	Length of fl-ORF	No. of valid sites	No. of seq.	Polymorphisms			No. of haplotypes	*H*	π	Tajima's *D*
						LoF	*Ns*	*S*				
HvWRKY1/38	*HORVU.MOREX.r2.6HG0471210.1*	*Hs*	1062	1046	75	0	0	7	8	0.69100	0.00090	-0.95917
		Hv			125	0	2	2	5	0.31900	0.00032	-0.93499
HvWRKY2	*HORVU.MOREX.r2.7HG0616250.1*	*Hs*	960	272	67	0	2	4	6	0.70200	0.00363	-0.44061
		Hv			117	0	3	3	6	0.68800	0.00377	0.80694
HvWRKY3	*HORVU.MOREX.r2.5HG0400750.1*	*Hs*	996	567	90	0	6	14	12	0.81300	0.00311	-1.48667
		Hv			134	0	6	9	7	0.57500	0.00260	-1.18422
HvWRKY4	*HORVU.MOREX.r2.2HG0091860.1*	*Hs*	1047	102	61	0	0	2	3	0.32700	0.00327	-1.34627
		Hv			130	0	3	1	4	0.17400	0.00220	-1.59618
HvWRKY5	*HORVU.MOREX.r2.3HG0222170.1*	*Hs*	714	649	84	0	5	1	7	0.46100	0.00081	-1.04538
		Hv			135	0	2	1	4	0.64400	0.00135	1.16844
HvWRKY6	*HORVU.MOREX.r2.7HG0577260.1*	*Hs*	1566	1373	90	0	5	4	8	0.26600	0.00022	-1.74566
		Hv			135	0	2	2	5	0.53200	0.00042	-0.23802
HvWRKY8	*HORVU.MOREX.r2.5HG0360410.1*	*Hs*	1068	832	88	0	2	7	7	0.35400	0.00061	-1.69853
		Hv			136	0	1	0	2	0.01500	0.00002	-1.21447
HvWRKY9	*HORVU.MOREX.r2.5HG0423130.1*	*Hs*	1188	1130	71	0	6	25	20	0.89200	0.00459	-0.51639
		Hv			119	0	3	12	6	0.66700	0.00444	2.94685*
HvWRKY10	*HORVU.MOREX.r2.2HG0130750.1*	*Hs*	957	106	89	0	1	2	4	0.39600	0.00391	-0.03032
		Hv			126	0	0	3	4	0.51100	0.00533	-0.537679
HvWRKY11	*HORVU.MOREX.r2.5HG0432220.1*	*Hs*	1359	766	67	0	11	10	17	0.81100	0.00304	-1.08243
		Hv			117	0	5	1	6	0.51600	0.00089	-1.00012

Genes	Gene IDs	Type	Length of fl-ORF	No. of valid sites	No. of seq.	Polymorphisms			No. of haplotypes	*H*	*π*	Tajima's *D*
						LoF	*Ns*	*S*				
HvWRKY12	*HORVU.MOREX.r2.2HG0079820.1*	*Hs*	591	537	54	0	6	19	26	0.96400	0.00573	-1.23073
		Hv			101	0	7	12	21	0.92800	0.00493	-0.84539
HvWRKY13	*HORVU.MOREX.r2.1HG0067480.1*	*Hs*	420	292	83	0	0	10	8	0.62200	0.00674	0.14762
		Hv			132	0	0	8	5	0.74100	0.00921	2.39787*
HvWRKY15	*HORVU.MOREX.r2.5HG0425110.1*	*Hs*	930	554	89	0	7	8	14	0.77800	0.00234	-1.54089
		Hv			135	0	3	5	6	0.54000	0.00213	-0.64344
HvWRKY17	*HORVU.MOREX.r2.1HG0023190.1*	*Hs*	600	521	88	0	3	2	7	0.25300	0.00051	-1.58417
		Hv			132	0	1	1	3	0.46600	0.00095	0.67992
HvWRKY18	*HORVU.MOREX.r2.3HG0236250.1*	*Hs*	633	307	83	0	5	6	8	0.30400	0.00152	-0.63467
		Hv			136	0	3	2	5	0.25200	0.00144	-0.53028
HvWRKY19	*HORVU.MOREX.r2.1HG0066230.1*	*Hs*	657	191	89	0	1	9	11	0.63000	0.00421	-0.56455
		Hv			136	0	1	1	3	0.31900	0.00249	0.16758
HvWRKY20	*HORVU.MOREX.r2.1HG0066270.1*	*Hs*	651	578	80	0	6	10	13	0.61500	0.00311	-1.07527
		Hv			132	0	4	2	3	0.19600	0.00123	-0.90743
HvWRKY22	*HORVU.MOREX.r2.5HG0392720.1*	*Hs*	999	729	69	0	12	7	14	0.83300	0.00245	-1.62753
		Hv			112	0	2	3	5	0.68300	0.00217	0.79454
HvWRKY23	*HORVU.MOREX.r2.7HG0616260.1*	*Hs*	1179	235	86	0	3	7	11	0.56500	0.00415	-1.49465
		Hv			131	0	1	6	5	0.56300	0.00530	-0.43439
HvWRKY26	*HORVU.MOREX.r2.1HG0074460.1*	*Hs*	765	445	46	0	2	3	6	0.52100	0.00133	-0.72485
		Hv			101	0	0	0	1	0	0	-1.17355
HvWRKY28	*HORVU.MOREX.r2.5HG0392730.1*	*Hs*	1041	869	44	0	6	4	9	0.52700	0.00095	-1.78499
		Hv			89	0	3	3	6	0.65700	0.00101	-0.20339

Genes	Gene IDs	Type	Length of fl-ORF	No. of valid sites	No. of seq.	Polymorphisms			No. of haplotypes	*H*	*π*	Tajima's *D*
						LoF	*Ns*	*S*				
HvWRKY31	*HORVU.MOREX.r2.1HG0057170.1*	*Hs*	930	877	73	0	19	16	18	0.89000	0.00600	-0.52370
		Hv			136	0	3	3	6	0.12800	0.00042	-1.35959
HvWRKY32	*HORVU.MOREX.r2.2HG0166600.1*	*Hs*	879	150	90	0	1	4	9	0.74600	0.00708	0.48328
		Hv			137	0	1	2	4	0.64000	0.00670	1.66345
HvWRKY33	*HORVU.MOREX.r2.4HG0316370.1*	*Hs*	621	49	90	0	1	2	4	0.35900	0.00751	-0.56955
		Hv			137	0	1	0	2	0.01500	0.00030	-0.91966
HvWRKY34	*HORVU.MOREX.r2.4HG0294290.1*	*Hs*	672	570	89	0	6	7	12	0.65000	0.00232	-1.08278
		Hv			137	0	2	1	2	0.07100	0.00037	-0.94982
HvWRKY35/41	*HORVU.MOREX.r2.2HG0106180.1*	*Hs*	1278	874	90	0	4	9	11	0.79200	0.00321	0.53148
		Hv			136	0	3	3	7	0.42700	0.00065	-0.84340
HvWRKY37	*HORVU.MOREX.r2.3HG0197030.1*	*Hs*	1485	201	90	0	4	3	8	0.26700	0.00141	-0.90985
		Hv			137	0	0	0	1	0	0	-0.16439
HvWRKY39	*HORVU.MOREX.r2.3HG0239890.1*	*Hs*	846	328	55	0	0	2	3	0.07200	0.00022	-0.65114
		Hv			101	0	0	1	2	0.02000	0.00006	-0.90236
HvWRKY40	*HORVU.MOREX.r2.5HG0386470.1*	*Hs*	1005	233	90	0	3	1	5	0.56600	0.00269	-0.18658
		Hv			136	0	2	0	3	0.02900	0.00013	-1.21447
HvWRKY42	*HORVU.MOREX.r2.5HG0405940.1*	*Hs*	2031	1918	82	0	11	24	28	0.93400	0.00171	-1.64952
		Hv			128	0	6	10	13	0.71500	0.00059	-1.61485
HvWRKY43	*HORVU.MOREX.r2.1HG0057680.1*	*Hs*	1539	302	85	0	5	6	12	0.76900	0.00378	-1.19480
		Hv			130	0	1	4	6	0.34000	0.00119	-0.50474
HvWRKY45	*HORVU.MOREX.r2.3HG0237940.1*	*Hs*	894	237	62	0	3	5	8	0.80900	0.00901	0.63440
		Hv			115	0	2	4	5	0.46200	0.00448	-0.25603

**Genes**	**Gene IDs**	**Type**	**Length of fl-ORF**	**No. of valid sites**	**No. of seq.**	**Polymorphisms**			**No. of haplotypes**	***H***	***π***	**Tajima's *D***
						LoF	*Ns*	*S*				
HvWRKY46	*HORVU.MOREX.r2.2HG0107970.1*	*Hs*	1848	1463	90	0	3	7	10	0.60200	0.00055	-1.30393
		Hv			137	0	2	5	7	0.32400	0.00035	-1.16404
HvWRKY47	*HORVU.MOREX.r2.3HG0209060.1*	*Hs*	1710	1547	58	0	3	5	9	0.55200	0.00042	-1.82310
		Hv			104	0	3	5	9	0.55200	0.00049	-0.92263
HvWRKY50	*HORVU.MOREX.r2.3HG0254080.1*	*Hs*	1677	627	70	0	2	15	19	0.84100	0.00260	-1.22615
		Hv			129	0	1	4	5	0.43200	0.00084	0.37584
HvWRKY51	*HORVU.MOREX.r2.3HG0230420.1*	*Hs*	1095	554	90	0	0	1	2	0.02200	0.00004	0.63797
		Hv			137	0	0	0	1	0	0	1.53987
HvWRKY52	*HORVU.MOREX.r2.5HG0400740.1*	*Hs*	996	595	82	0	10	10	17	0.86000	0.00404	-0.81277
		Hv			132	0	6	7	11	0.76100	0.00418	-0.03733
HvWRKY55/87	*HORVU.MOREX.r2.1HG0074420.1*	*Hs*	1044	337	74	0	9	6	14	0.78700	0.00576	-1.64938
		Hv			120	0	2	0	3	0.03300	0.00010	-1.30200
HvWRKY56	*HORVU.MOREX.r2.1HG0076100.1*	*Hs*	930	187	90	0	4	9	13	0.64600	0.00903	-0.61533
		Hv			137	0	2	7	7	0.49100	0.00779	0.29297
HvWRKY57	*HORVU.MOREX.r2.4HG0313700.1*	*Hs*	1119	844	90	0	7	3	9	0.64800	0.00113	-1.14242
		Hv			137	0	2	1	3	0.02900	0.00004	-1.34929
HvWRKY58	*HORVU.MOREX.r2.4HG0284540.1*	*Hs*	1023	881	90	0	8	21	25	0.92700	0.00460	-0.60447
		Hv			137	0	6	10	12	0.83600	0.00539	1.76245
HvWRKY59	*HORVU.MOREX.r2.2HG0144420.1*	*Hs*	2211	843	61	0	6	14	15	0.80800	0.00535	-0.21297
		Hv			103	0	2	8	7	0.34400	0.00113	-1.54822
HvWRKY60	*HORVU.MOREX.r2.7HG0539640.1*	*Hs*	1830	987	63	0	13	16	21	0.91000	0.00515	-0.08586
		Hv			105	0	9	11	12	0.82000	0.00361	0.41921

Genes	Gene IDs	Type	Length of fl-ORF	No. of valid sites	No. of seq.	Polymorphisms			No. of haplotypes	*H*	*π*	Tajima's *D*
						LoF	*Ns*	*S*				
HvWRKY61	*HORVU.MOREX.r2.5HG0371550.1*	*Hs*	1479	736	90	0	0	4	5	0.14900	0.00024	-1.35226
		Hv			136	0	0	0	1	0	0	NA
HvWRKY64	*HORVU.MOREX.r2.3HG0226640.1*	*Hs*	957	807	90	0	0	1	2	0.06500	0.00008	-0.64457
		Hv			137	0	2	1	4	0.50100	0.00069	0.13746
HvWRKY65	*HORVU.MOREX.r2.5HG0425470.1*	*Hs*	1260	355	85	0	3	8	11	0.70200	0.00322	-0.52030
		Hv			128	0	0	3	4	0.57400	0.00182	0.58766
HvWRKY66	*HORVU.MOREX.r2.6HG0513200.1*	*Hs*	1692	533	77	0	7	8	12	0.76500	0.00367	-0.42524
		Hv			132	0	2	3	4	0.43700	0.00175	0.49741
HvWRKY67	*HORVU.MOREX.r2.5HG0400720.1*	*Hs*	549	524	82	0	7	10	14	0.82600	0.00567	-0.35721
		Hv			126	0	2	6	6	0.66500	0.00378	1.14241
HvWRKY68	*HORVU.MOREX.r2.2HG0153650.1*	*Hs*	693	588	75	0	4	5	11	0.79000	0.00222	-0.88398
		Hv			127	0	0	2	3	0.47100	0.00083	-0.19483
HvWRKY69	*HORVU.MOREX.r2.1HG0073000.1*	*Hs*	1230	223	88	0	1	9	10	0.53400	0.00313	0.19644
		Hv			135	0	0	1	2	0.12500	0.00056	0.42403
HvWRKY70	*HORVU.MOREX.r2.3HG0247840.1*	*Hs*	825	149	78	0	2	3	6	0.30200	0.00298	-1.67988
		Hv			128	0	2	0	3	0.09100	0.00103	-0.66546
HvWRKY71	*HORVU.MOREX.r2.7HG0584730.1*	*Hs*	1026	709	87	0	10	5	14	0.58200	0.00152	-1.75770
		Hv			129	0	7	1	7	0.64600	0.00140	0.21203
HvWRKY73	*HORVU.MOREX.r2.5HG0446310.1*	*Hs*	1065	699	87	0	13	10	11	0.62200	0.00428	-0.92155
		Hv			134	0	8	6	7	0.15700	0.00071	-2.15932*
HvWRKY74	*HORVU.MOREX.r2.1HG0042200.1*	*Hs*	1023	924	74	0	6	9	23	0.92700	0.00253	-0.51567
		Hv			118	0	3	4	7	0.59200	0.00219	0.84291

Genes	Gene IDs	Type	Length of fl-ORF	No. of valid sites	No. of seq.	Polymorphisms			No. of haplotypes	*H*	*π*	Tajima's *D*
						LoF	*Ns*	*S*				
HvWRKY75	*HORVU.MOREX.r2.6HG0497280.1*	*Hs*	684	492	90	0	6	6	11	0.69500	0.00241	-1.19886
		Hv			135	0	6	3	9	0.59300	0.00171	-1.08275
HvWRKY76	*HORVU.MOREX.r2.3HG0273730.1*	*Hs*	1155	341	66	0	4	2	8	0.72200	0.00316	-0.72597
		Hv			112	0	2	2	3	0.63600	0.00296	0.45090
HvWRKY79	*HORVU.MOREX.r2.2HG0129050.1*	*Hs*	858	505	82	0	9	14	17	0.87100	0.00704	-0.32834
		Hv			130	0	6	4	7	0.35000	0.00261	-0.48211
HvWRKY80	*HORVU.MOREX.r2.5HG0384210.1*	*Hs*	1014	665	88	0	14	7	20	0.86700	0.00318	-1.47988
		Hv			136	0	2	5	5	0.65800	0.00245	0.75630
HvWRKY83	*HORVU.MOREX.r2.2HG0083410.1*	*Hs*	867	563	60	0	25	19	16	0.84500	0.01101	-0.65394
		Hv			103	0	19	14	11	0.73700	0.00831	-0.44455
HvWRKY89	*HORVU.MOREX.r2.3HG0226770.1*	*Hs*	1011	926	86	0	4	8	13	0.31900	0.00037	-2.08241*
		Hv			136	0	0	2	3	0.50000	0.00059	0.69940
HvWRKY93	*HORVU.MOREX.r2.5HG0384220.1*	*Hs*	924	629	88	0	13	4	9	0.53900	0.00581	0.37261
		Hv			136	0	11	3	5	0.35800	0.00567	0.92507
HvWRKY95	*HORVU.MOREX.r2.1HG0057190.1*	*Hs*	903	599	82	0	19	6	19	0.86100	0.00714	0.02828
		Hv			134	0	9	3	4	0.07300	0.00085	-1.79431*
HvWRKY96	*HORVU.MOREX.r2.5HG0389690.1*	*Hs*	546	362	77	0	13	10	22	0.83400	0.00553	-1.74628
		Hv			115	0	7	5	12	0.71700	0.00636	-0.41557
HvWRKY101	*HORVU.MOREX.r2.3HG0238260.1*	*Hs*	696	489	58	0	5	7	14	0.74700	0.00252	-0.89318
		Hv			116	0	0	2	3	0.06800	0.00014	-1.34749
HvWRKY102	*HORVU.MOREX.r2.2HG0161240.1*	*Hs*	1536	1097	82	0	6	11	17	0.75500	0.00109	-1.49867
		Hv			126	0	1	1	3	0.53400	0.00053	1.56650

**Genes**	**Gene IDs**	**Type**	**Length of fl-ORF**	**No. of valid sites**	**No. of seq.**	**Polymorphisms**			**No. of haplotypes**	***H***	***π***	**Tajima's *D***
						LoF	*Ns*	*S*				
HvWRKY105	*HORVU.MOREX.r2.2HG0091840.1*	*Hs*	1059	432	70	0	11	5	13	0.74800	0.00463	0.04064
		Hv			129	0	8	3	7	0.61100	0.00395	-0.42878

Short reads, which were retrieved from the publically-released exome sequencing of 90 wild barley and 137 landraces (Russell et al. 2016), were subjected for mapping and SNP calling. The accessions showing sequences with missing or heterozygote were excluded for analysis. Loss-of-function variation (*LoF*), non-synonymous (*Ns*), synonymous (*S*), haplotype diversity (*H*), nucleotide diversity (π). The parameters (*H*, π) are calculated using DnaSP v5.10.01 with the gaps not-considered algorithm. The Tajima's *D* in the coding sequences of *WRKYs* was performed by using VCFtools v0.1.13. [*]*WRKY* genes with selection signature by considering the 2.5% and 97.5% quantiles of the Tajima's *D* distribution. NA, not determined.
