# Supplementary material for: Genome-Wide Characterization of WRKY Transcription Factors Revealed Gene Duplication and Diversification in Populations of Wild to Domesticated Barley
Source: Int J Mol Sci. 2021 May 19;22(10):5354. doi: 10.3390/ijms22105354 (PMC8160967; doi:10.3390/ijms22105354)
Supplement: Supplementary file 1 [file ijms-22-05354-s001.zip › Table S8-The primers used in this study.pdf]

**Table S8.** The primers used in this study

| primer | Sequence (5′-3′) |
|---|---|
| WRKY9-Gateway-F | GGGGACAAGTTTGTACAAAAAAGCAGGCTTTATGGATGGCATGGAGGAATCC |
| WRKY9-Gateway-R | GGGGACCACTTTGTACAAGAAAGCTGGGTTGGCTGGTTGGGCTGCTGC |
