# Supplementary material for: Genome-Wide Characterization of WRKY Transcription Factors Revealed Gene Duplication and Diversification in Populations of Wild to Domesticated Barley
Source: Int J Mol Sci. 2021 May 19;22(10):5354. doi: 10.3390/ijms22105354 (PMC8160967; doi:10.3390/ijms22105354)
Supplement: Supplementary file 1 [file ijms-22-05354-s001.zip › Supplementary_Material_Figures.pdf]

Supplementary Materials

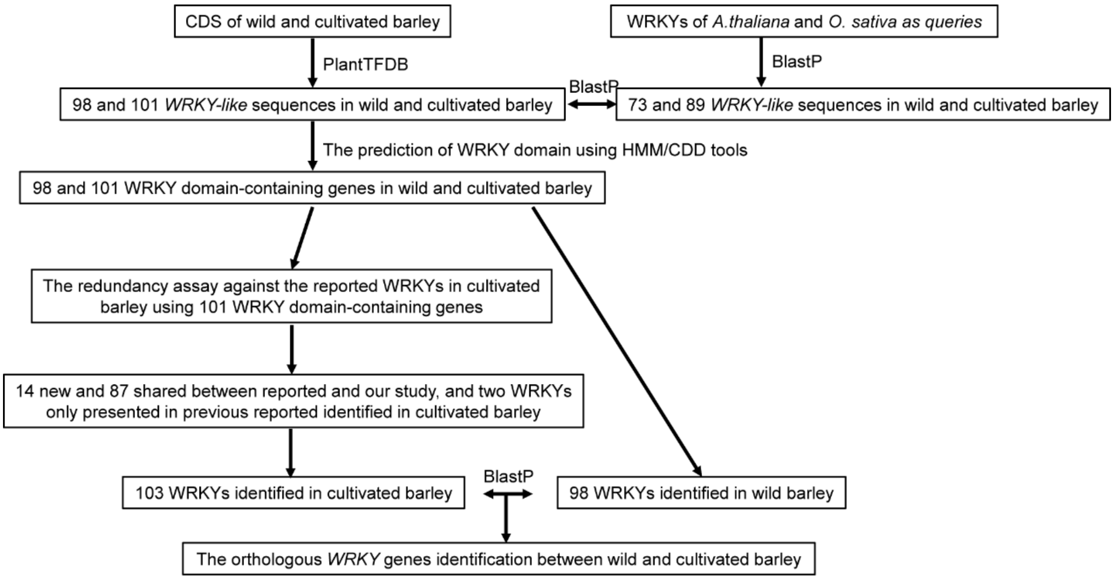

Figure S1. The workflow of identification of *WRKYs* in wild and cultivated barley.

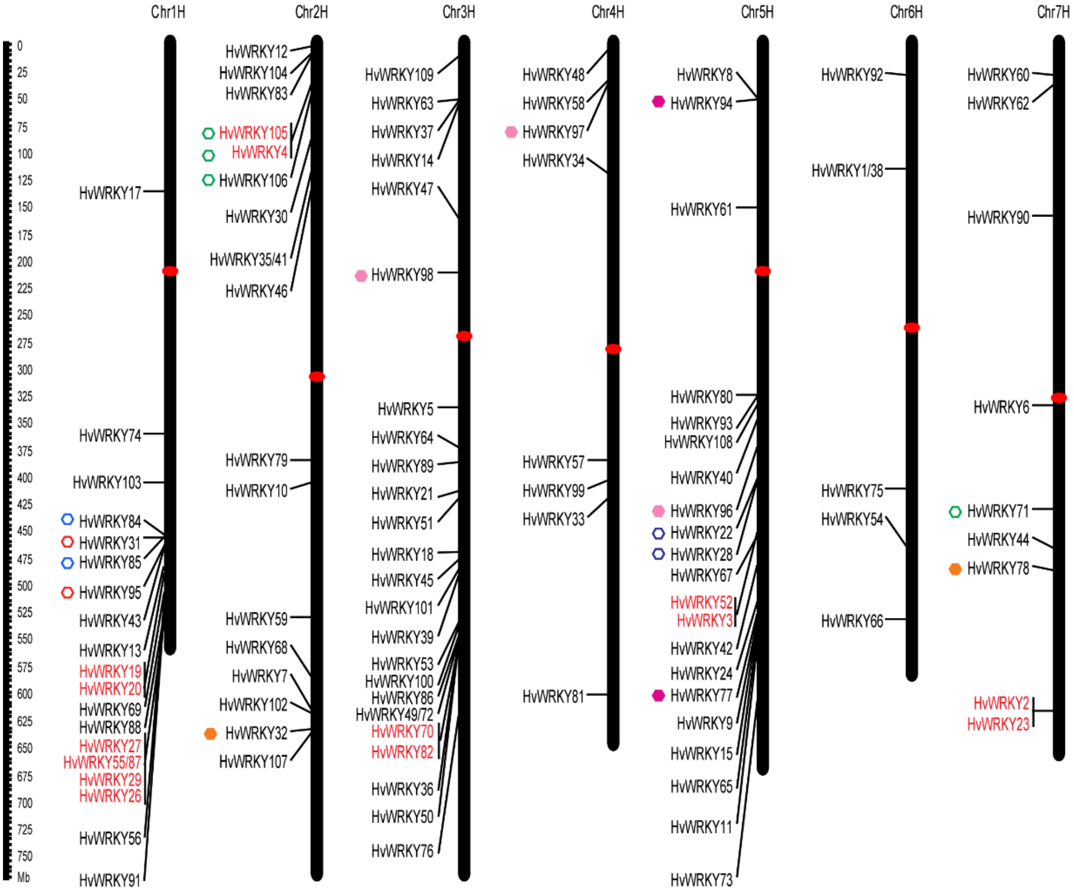

Figure S2. Chromosomal locations of *HvWRKYs*. The chromosomal position of each *HvWRKY* was mapped according to the barley genome Morex_v2. The

chromosome numbers were shown at the top of each chromosome. The centromere locations were indicated by red solid ellipse, and the location of each *WRKY* gene was indicated by a line. The length refers to the size of the chromosome. Tandem duplicated genes were indicated with red fonts, and segmentally duplicated genes were shown with hexagons of different colors.

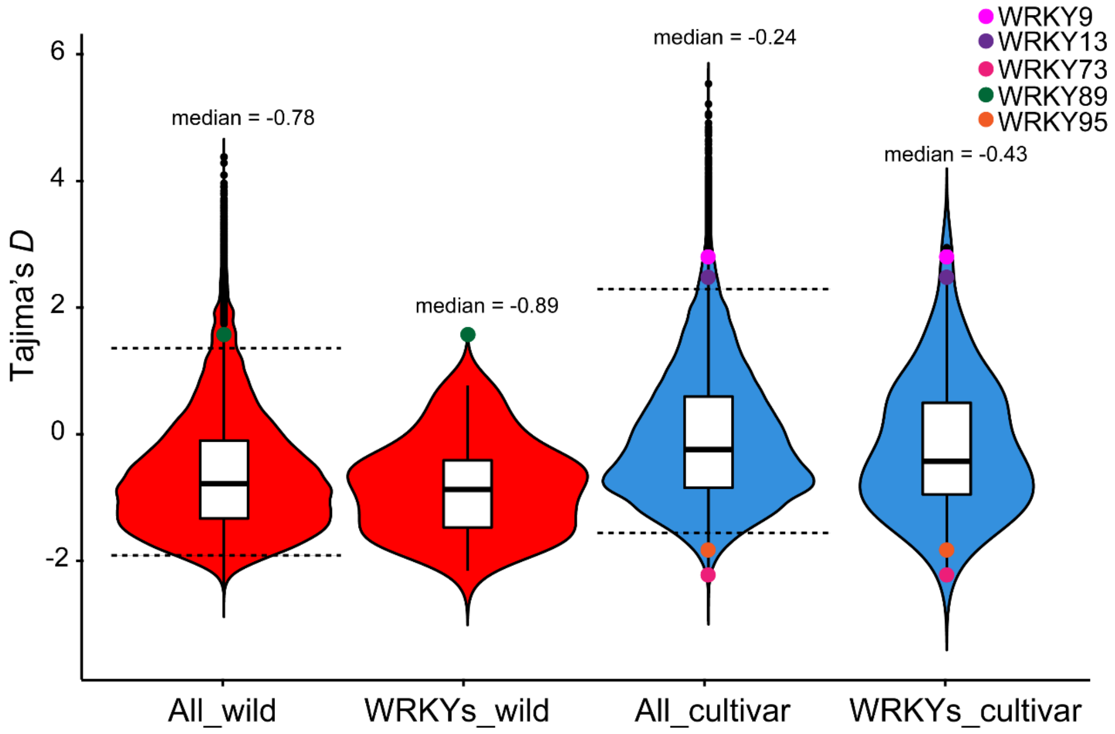

Figure S3. Violinplot of the Tajima's *D* of all genes and *WRKY*s in wild and cultivated barley population. Red and blue represented wild and cultivated barley, respectively. All_wild and All_cultivar indicated all genes from wild and cultivated barley populations, respectively, while WRKYs_wild and WRKYs_cultivar denoted all *WRKYs* from wild and cultivated barley populations, respectively. The dotted lines showed the 97.5% and 2.5% quantiles in each of barley compartments. *WRKYs* underwent selection were indicated with the solid colored dots.
