# Supplementary material for: Genome-Wide Characterization of WRKY Transcription Factors Revealed Gene Duplication and Diversification in Populations of Wild to Domesticated Barley
Source: Int J Mol Sci. 2021 May 19;22(10):5354. doi: 10.3390/ijms22105354 (PMC8160967; doi:10.3390/ijms22105354)
Supplement: Supplementary file 1 [file ijms-22-05354-s001.zip › Table S3-Presence and position of conserved domains identified within barley.pdf]

Table S3. Presence and position of conserved domains identified within barley WRKY proteins using HMMER

genes	WRKY domain	Plant_Zn _clust	NB-ARC	Rx_N	VQ	RVT_2	Retrotran_gag_ 2	Gag_pre -integrs
HvWRKY1/38	195-253							
HvWRKY2	183-241							
HvWRKY3	172-230							
HvWRKY4	121-181							
HvWRKY5	117-174							
HvWRKY6	199-255							
	367-424							
HvWRKY7	372-430	188-230						
		327-369						
HvWRKY8	288-346	242-285						
HvWRKY9	329-387	282-326						
HvWRKY10	247-305	195-244						
HvWRKY11	380-438	331-377						
HvWRKY12	104-161							
HvWRKY13	61-118							
HvWRKY14	188-145							
HvWRKY15	138-195							
HvWRKY17	109-166							
HvWRKY18	126-183							
HvWRKY19	131-188							
HvWRKY20	129-186							
HvWRKY21	126-186							
HvWRKY22	122-182							
HvWRKY23	248-106							
HvWRKY24	1377-1437		481-725	304-415				
	1492-1552							
HvWRKY26	95-155							
HvWRKY27	104-164							
HvWRKY28	124-184							
HvWRKY29	97-157							
HvWRKY30	120-180							
HvWRKY31	113-173							
HvWRKY32	107-167							
HvWRKY33	33-94							
HvWRKY34	43-104							
HvWRKY35/41	154-209							
	320-377							
HvWRKY36	168-225							
HvWRKY37	241-199							
HvWRKY39	79-137							

genes	WRKY domain	Plant_Zn_clust	NB-ARC	Rx_N	VQ	RVT_2	Retrotran_gag_2	Gag_pre-integrs
HvWRKY40	116-172							
	276-331							
HvWRKY42	276-332							
	489-546							
HvWRKY43	178-234							
	345-402							
HvWRKY44	162-221							
HvWRKY45	74-132							
HvWRKY46	231-287							
	405-462							
HvWRKY47	301-359							
HvWRKY48	34-90							
HvWRKY49/72	124-188							
HvWRKY50	215-171							
	375-432							
HvWRKY51	168-225							
HvWRKY52	172-230							
HvWRKY53	49-110							
HvWRKY54	235-293							
HvWRKY55/87	106-166							
HvWRKY56	167-224							
HvWRKY57	89-145							
	257-314							
HvWRKY58	273-331	225-270						
HvWRKY59	300-356							
	530-587							
HvWRKY60	232-290							
HvWRKY61	224-280							
	381-438							
HvWRKY62	140-200							
HvWRKY63	131-188							
HvWRKY64	157-215							
HvWRKY65	185-241							
	313-370							
HvWRKY66	318-376							
HvWRKY67	26-84							
HvWRKY68	153-210							
HvWRKY69	203-260							
HvWRKY70	105-164							
HvWRKY71	116-174							
HvWRKY73	173-231							
HvWRKY74	182-240							

genes	WRKY domain	Plant_Zn_clust	NB-ARC	Rx_N	VQ	RVT_2	Retrotran_gag_2	Gag_pre-integrs
HvWRKY75	153-210							
HvWRKY76	152-209							
HvWRKY77	233-291	185-230						
HvWRKY78	112-172							
HvWRKY79	127-184							
HvWRKY80	133-198							
HvWRKY81	140-198							
HvWRKY82	103-162							
HvWRKY83	107-172							
HvWRKY84	135-196							
HvWRKY85	138-199							
HvWRKY86	120-183							
HvWRKY88	335-393							
HvWRKY89	188-218							
HvWRKY90	247-303<br>443-500							
HvWRKY91	156-214							
HvWRKY92	202-262							
HvWRKY93	142-210							
HvWRKY94	220-278	168-215						
HvWRKY95	105-165							
HvWRKY96	108-130	61-105						
HvWRKY97	108-130	59-105						
HvWRKY98	108-130	63-105						
HvWRKY99	113-173							
HvWRKY100	67-128							
HvWRKY101	153-210							
HvWRKY102	243-301							
HvWRKY103	174-234							
HvWRKY104	127-194							
HvWRKY105	119-179							
HvWRKY106	121-181							
HvWRKY107	124-187							
HvWRKY108	133-206							
HvWRKY109	80-140							
HsWRKY1	195-253							
HsWRKY2	183-241							
HsWRKY3	166-224							
HsWRKY4	121-181							
HsWRKY5	117-203							
HsWRKY6	754-810<br>922-979					537-656	95-235	373-440

genes	WRKY domain	Plant_Zn _clust	NB-ARC	Rx_N	VQ	RVT_2	Retrotran_gag_2	Gag_pre -integrs
HsWRKY7	233-291							
HsWRKY8	288-346	242-285						
HsWRKY9	327-385	280-324						
HsWRKY10	247-305	195-244						
HsWRKY11	380-438	331-377						
HsWRKY12	104-161							
HsWRKY13	143-200							
HsWRKY14	171-228							
HsWRKY15	138-195							
HsWRKY17	109-166							
HsWRKY18	124-181							
HsWRKY19	131-188							
HsWRKY20	129-186							
HsWRKY21	126-186							
HsWRKY22	122-182							
HsWRKY23	195-253							
HsWRKY24	1294-1354		398-642	221-332				
	1409-1469							
HsWRKY26	95-155							
HsWRKY27	104-164							
HsWRKY28	124-184							
HsWRKY30	120-180							
HsWRKY32	105-127							
HsWRKY33	33-94							
HsWRKY34	43-104							
HsWRKY36	168-225							
HsWRKY37	241-299							
HsWRKY39	79-137							
HsWRKY40	126-182							
	286-341							
HsWRK35/41	218-273							
	384-441							
HsWRKY42	276-332							
	489-546							
HsWRKY43	178-234							
	345-402							
HsWRKY44	162-221							
HsWRKY45	74-132							
HsWRKY46	231-287							
	405-462							
HsWRKY47	301-359							
HsWRKY48	34-90							

genes	WRKY domain	Plant_Zn _clust	NB-ARC	Rx_N	VQ	RVT_2	Retrotran_gag_ 2	Gag_pre -integrs
HsWRKY49/72	124-188							
HsWRKY50	215-271							
	375-432							
HsWRKY51	168-225							
HsWRKY53	49-110							
HsWRKY54	235-293							
HsWRKY55	106-166							
HsWRKY56	163-220							
HsWRKY57	105-161							
	273-330							
HsWRKY58	273-331	225-270						
HsWRKY59	304-360							
	534-591							
HsWRKY60	236-294							
HsWRKY61	224-280							
	381-405							
HsWRKY62	140-200							
HsWRKY63	131-188							
HsWRKY64	150-208							
HsWRKY65	185-241							
	337-360							
HsWRKY66	318-376							
HsWRKY67	128-186							
HsWRKY68	116-173							
HsWRKY69	203-260							
HsWRKY70	105-164							
HsWRKY71	116-174							
HsWRKY73	173-231							
HsWRKY74	182-240							
HsWRKY75	141-198							
HsWRKY76	149-206							
HsWRKY77	235-293	187-232						
HsWRKY78	204-264							
HsWRKY79	127-184							
HsWRKY80	133-198							
HsWRKY81	140-198							
HsWRKY82	103-162							
HsWRKY83	110-175							
HsWRKY84	135-196							
HsWRKY88	380-438							
HsWRKY89	188-245							

genes	WRKY domain	Plant_Zn _clust	NB-ARC	Rx_N	VQ	RVT_2	Retrotran_gag_ 2	Gag_pre -integrs
HsWRKY90	230-286							
	426-483							
HsWRKY91	156-214							
HsWRKY93	148-216							
HsWRKY94	220-278	168-215						
HsWRKY95	105-165							
HsWRKY99	83-143							
HsWRKY100	67-128							
HsWRKY101	82-139							
HsWRKY102	243-301							
HsWRKY104	116-183							
HsWRKY105	119-179							
HsWRKY108	133-206							
HsWRKY109	411-471							
HsWRKY110	108-130	63-105						
HsWRKY111	108-128	63-105						
HsWRKY112	108-135	62-105						
HsWRKY113	23-89							
HsWRKY114	8-65							
HsWRKY115	1264-1325		379-633	196-316	58-85			
HsWRKY116	122-182							
	267-327							
